# Enhancer of zeste 2 polycomb repressive complex 2 subunit overexpression suppresses apoptosis in canine T-cell lymphoma: an *in vitro* functional analysis

**DOI:** 10.3389/fvets.2026.1823343

**Published:** 2026-06-12

**Authors:** Honoka Kawamura, Rina Ishikawa-Shinohara, Asuka Okamura, Susumu Kohno, Viviana Gonzalez-Astudillo, Ayako Wakabayashi, Yukiko Aikawa, Issay Kitabayashi, Takuya Mizuno, Noriko Gotoh, Masaki Michishita, Yukino Machida

**Affiliations:** 1 Department of Veterinary Pathology, Nippon Veterinary and Life Science University, Tokyo, Japan; 2 Division of Oncology and Molecular Biology, Cancer Research Institute, Kanazawa University, Kanazawa, Japan; 3 School of Veterinary Science, The University of Queensland, Gatton, QLD, Australia; 4 Department of Microbiology and Immunology, Nippon Medical School, Tokyo, Japan; 5 Department of Immune Medicine, National Cancer Center Research Institute, Tokyo, Japan; 6 Oncology Innovation Center/Center for Translational Research, Fujita Health University, Toyoake, Aichi, Japan; 7 Laboratory of Molecular Diagnostics and Therapeutics, Department of Veterinary Medicine, Joint Faculty of Veterinary Medicine, Yamaguchi University, Yoshida, Yamaguchi Prefecture, Japan; 8 Division of Cancer Cell Biology, Cancer Research Institute, Institute for Frontier Science Initiative, Kanazawa University, Kanazawa, Japan; 9 Environmental Stress Research Center (eSRC), Kanazawa University, Kanazawa, Japan; 10 Center for One Health, One Welfare, Nippon Veterinary and Life Science University, Tokyo, Japan; 11 Laboratory of Veterinary Pathology, Cooperative Department of Veterinary Medicine, Tokyo University of Agriculture and Technology, Tokyo, Japan

**Keywords:** apoptosis, dog, enhancer of zeste 2 polycomb repressive complex 2 subunit, epigenetics, lymphoma

## Abstract

Lymphoma is one of the most common neoplastic diseases in dogs, and T-cell lymphoma in particular has frequently been reported to show limited sensitivity to conventional chemotherapy from the onset of treatment when compared with B-cell lymphoma. Owing to its high-grade and aggressive clinical behavior, there is a strong need to explore novel therapeutic approaches for canine T-cell lymphoma, including strategies targeting aberrant gene regulation. In this study, we focused on the *epigenetic enzyme enhancer of zeste 2 polycomb repressive complex 2 subunit (EZH2)* and investigated its functional role in canine T-cell lymphoma. The *EZH2* gene was ectopically introduced into canine T-cell lymphoma cell lines using a lentiviral vector system, and colony-forming ability, and apoptosis rate were evaluated. *EZH2* overexpression significantly enhanced colony-forming capacity, suppressed apoptosis, and increased the number of viable cells compared with control cells. These findings indicate that elevated *EZH2* expression confers a survival advantage to canine T-cell lymphoma cells. The present study provides novel evidence that overexpression of *EZH2* itself promotes tumor cell survival and aggressiveness. Our results suggest that *EZH2* may play a functional role in the maintenance and progression of at least a subset of canine T-cell lymphoma and may represent a potential driver of tumor malignancy. These findings contribute to a deeper understanding of lymphomagenesis and support the potential of *EZH2* as a therapeutic target in canine T-cell lymphoma.

## 1 Introduction

Lymphoma ranks third among the most common neoplastic diseases in dogs. Approximately 70–85% of cases are systemic and high grade, greatly reducing the chance of therapeutic interventions and thus prognosis and with treatment typically involving combination or single-agent chemotherapy as the first-line option. However, even when temporary remission is achieved, recurrence is common, and response duration is short even in cases responsive to rescue therapy. When considering all subtypes, T-cell lymphomas frequently show limited sensitivity to conventional chemotherapy from the onset of treatment, in comparison to more common subtypes such as B-cell lymphoma (1). The frequent high-grade aggression of certain canine tumors such as T-cell lymphomas grants the exploration of alternative therapeutical approaches, especially those targeting aberrant gene regulation.

Epigenetics refers to regulatory mechanisms, including DNA methylation and histone modifications, which control gene expression without altering the DNA sequence, playing a critical role in cancer development and progression (2). In the medical field, enhancer of zeste 2 polycomb repressive complex 2 subunit (EZH2) has gained attention as an epigenetic regulator selected by molecular targeted therapies. EZH2 is a key protein constituting polycomb repressive complex 2 (PRC2), a complex involved in regulating differentiation and the cell cycle. Specifically, accumulation of Tri-Methyl-Histone H3 (Lys27) trimethylate (H3K27me3), generated by trimethylation via EZH2, is thought to enhance cell proliferation by suppressing the expression of tumor suppressor genes (3). Moreover, highly activating *EZH2* mutations are frequently identified at early stages of tumorigenesis (4), suggesting that aberrantly activated PRC2 functions as a cancer driver. Consequently, EZH2 is recognized as an important tumorigenic factor, expressed in many human tumors, granting the development of EZH2-targeted epigenetic therapies.

While previous studies have reported EZH2 overexpression in multiple canine tumors, including lymphoma, it remains unclear whether this overexpression represents a mere epiphenomenon of malignant transformation or whether EZH2 functions as a key driver that actively contributes to tumor aggressiveness in canine lymphoma (5). Canine lymphoma closely resembles human non-Hodgkin lymphoma in terms of clinical behavior, histopathology, and molecular characteristics, and is therefore considered an excellent spontaneous animal model for the human non-Hodgkin lymphoma (1). Elucidating the shared tumor biological mechanisms between canine and human lymphoma has the potential to advance both human medicine and veterinary oncology. In humans, several studies in hematologic malignancies and various solid tumors have shown that EZH2 inhibition induces growth suppression, apoptosis, and immune activation (3, 6, 7). However, despite the frequent overexpression of EZH2 in tumors, relatively few studies have directly examined the functional consequences of EZH2 overexpression itself. In this study, we investigated the effects of EZH2 overexpression in canine T-cell lymphoma by ectopically introducing the *EZH2* gene into canine T-cell lymphoma cell lines and evaluated phenotypic changes to analyze the function of EZH2 as a novel therapeutic target candidate.

## 2 Methods

### 2.1 Cell line

A canine T-cell lymphoma cell line, CLC, was provided by the Laboratory of Veterinary Internal Medicine, The United Graduate School of Veterinary Science, Yamaguchi University, where this cell line was originally derived, established, and cultured previously (8). CLC was originally established from a high-grade lymphoma in an eight-year-old female French Bulldog. The cells showed a CD3-positive/CD21-negative phenotype by flow cytometry and were negative for *TCRγ* and *IgH* rearrangements by PARR analysis (8). CLC cells were maintained in R10 complete medium [RPMI-1640 with L-Glutamine and Phenol Red (189–02025, Fujifilm Wako, Osaka, Japan) with 10% fetal bovine serum (FBS, A5256801, Invitrogen, Waltham, MA, United States), antibiotic-antimycotic mixed stock solution (09366–44, Nacalai Tesque, Kyoto, Japan) and 55 μM 2-mercaptoethanol (131–14,572, Fujifilm Wako)]. HEK293T cells (Lenti- X 293 T Cell Line) were purchased from Clontech (Mountain View, CA, United States) and maintained in Dulbecco’s modified Eagle medium (DMEM) and high glucose (043–30,085, Fujifilm Wako). Cells were supplemented with 10% FBS (Invitrogen), antibiotic-antimycotic mixed stock solution (Nacalai Tesque), and incubated at 37 °C in an atmosphere containing 5% CO_2_.

### 2.2 Cell line authentication statement

CLC cells were maintained at the Laboratory of Veterinary Internal Medicine, the United Graduate School of Veterinary Science, Yamaguchi University. The laboratory where the CLC cells were sourced from confirmed the animal species of origin were dogs (8).

### 2.3 Lentiviral vectors for lentiviral construction

First-strand cDNA library was synthesized from MDCK cell mRNA by PrimeScrip II 1st strand cDNA Synthesis Kit (6210A, TAKARA Bio, Kyoto, Japan), according to manufacturer’s instructions. The *Canis familiaris EZH2* gene was amplified by PCR using the forward primer 5’-GGGGACAAGTTTGTACAAAAAAGCAGGC TTCG CCACCATGGACTACAAAGACGATGACGACAAGATGGG CCAGACTGGGAAGAAA-3′ and the reverse primer 5’-GGGGACC AC TTTGTACAAGAAAGCTGGGTTTCAAGGGATTTCCATCT CTCTTTCG-3′. The EGFP gene was amplified from pEGFP-C1 using the forward primer 5’-AAAAAGCAGGCTTAATGGTGAGCAAGG GCGAG-3′, the reverse primer 5’-AGAAAGCTGGGTTTTACTTG TACAGCTCGTCCATGCC-3′. The PCR products were cloned into pDONR223 and subsequently transferred into pLX302 (#25896, Addgene, Watertown, MA, United States) via the LR clonase reaction. We used pCMV-VSV-G-RSV-Rev and pCAG- HIVgp as packaging plasmids, which were gifted by H. Miyoshi (RIKEN, Tsukuba, Japan).

### 2.4 Transduction of cells with lentiviral vectors

A culture medium containing virus was produced by co-transfecting plasmids in HEK293T cells using lipofectamine® 3,000 (L3000015, Thermo Fisher Scientific, Waltham, MA, United States) and Plus reagent (11,514,015, Thermo Fisher Scientific), as previously described (9). CLC cells (5 × 10^5^ cells/well) were plated in a 6-well plate and then cultured with 20% viral solution in DMEM (5 mL) and incubated overnight at 37 °C in 5% CO_2_. For selection of cells stably expressing EZH2, CLC cells were cultured in a medium containing 2.0 μg/mL puromycin. The recombinant DNA experiments were performed with the approval of the Nippon Veterinary and Life Science University Genetic Recombination Experiments Committee.

### 2.5 Colony assay using methylcellulose medium

Using methylcellulose medium, we cultured *EZH2*-transfected cells and control cells at the same cell density for the same duration. We then measured colony numbers to evaluate whether differences in colony formation occurred between the two cell types in medium. Cells were seeded at 1.0 × 10^4^ cells/well in 6-well plates containing 2 mL/well of MethoCult™ M3234 medium (03234, STEMCELL Technologies, Vancouver, BC, Canada) and 0.5 mL/well of RPMI medium. Cells were cultured for 4 days at 37 °C, 5% CO₂. Colony numbers in the Methocult medium were counted and measured under an optical microscope. Colonies were subjectively counted as either compact colonies with relatively high cell density or loose colonies with low cell density. Cultured cells in Methocult medium were recovered by washing with PBS and centrifugation (800 × g, 10 min). A total of three measurements were performed.

### 2.6 Detection of apoptosis by flow cytometry

To evaluate apoptotic cells following *EZH2* gene transfer, the MEBCYTO Apoptosis Kit (4,700, Medical & Biological Laboratories, Tokyo, Japan) was used with Annexin V- FITC and propidium iodide (PI) staining. Initially, 2 × 10^5^ cells were suspended in 85 μL of 1 × binding buffer, followed by the addition of 10 μL Annexin V- FITC and 5 μL PI. The mixture was then incubated at 25 °C for 15 min in the dark. The cells were then analyzed using a CytoFLEX flow cytometer (Beckman Coulter, Brea, CA, United States), and the data were processed using FlowJo v10.10.0 (BD Biosciences, Franklin Lakes, NJ, United States). Fluorescence compensation was performed using the following single-stained samples: (1) unstained cells, (2) Annexin V-FITC-stained cells, and (3) Propidium Iodide (PI)-stained cells. Annexin V (+)/PI (−) staining was indicative of early apoptosis. The apoptosis index was calculated as follows: Apoptosis index (%) = Annexin V- single- positive cells (%) + Annexin V/PI double- positive cells (%).

### 2.7 Statistical analysis

Colony counts are presented as the mean ± standard deviation. For the initial analysis, loose, dense, and total colony numbers were compared between CLC-control and CLC-EZH2 cells using unpaired two-tailed statistical tests. To further evaluate the effects of EZH2 overexpression while accounting for colony type and within-well dependency, colony counts were additionally analyzed using a mixed-effects two-way ANOVA, with vector (CLC-control vs. CLC-EZH2) and colony type (loose vs. dense) as fixed effects, with well included as a random effect. *Post–hoc* comparisons were performed using Sidak’s multiple comparisons test. To stabilize variance, colony counts were also analyzed after log-transformation. Degrees of freedom were estimated using the Kenward–Roger method. Differences in apoptotic cell populations between groups were analyzed using an unpaired two-tailed Student’s t-test after confirming homogeneity of variance. R (version 4.3.1) was used for statistical analysis. The significance level was set at 5% for all analyses.

## 3 Results

### 3.1 EZH2 is expressed in canine lymphoma cell lines following lentiviral gene transfer

**Figure 1:**
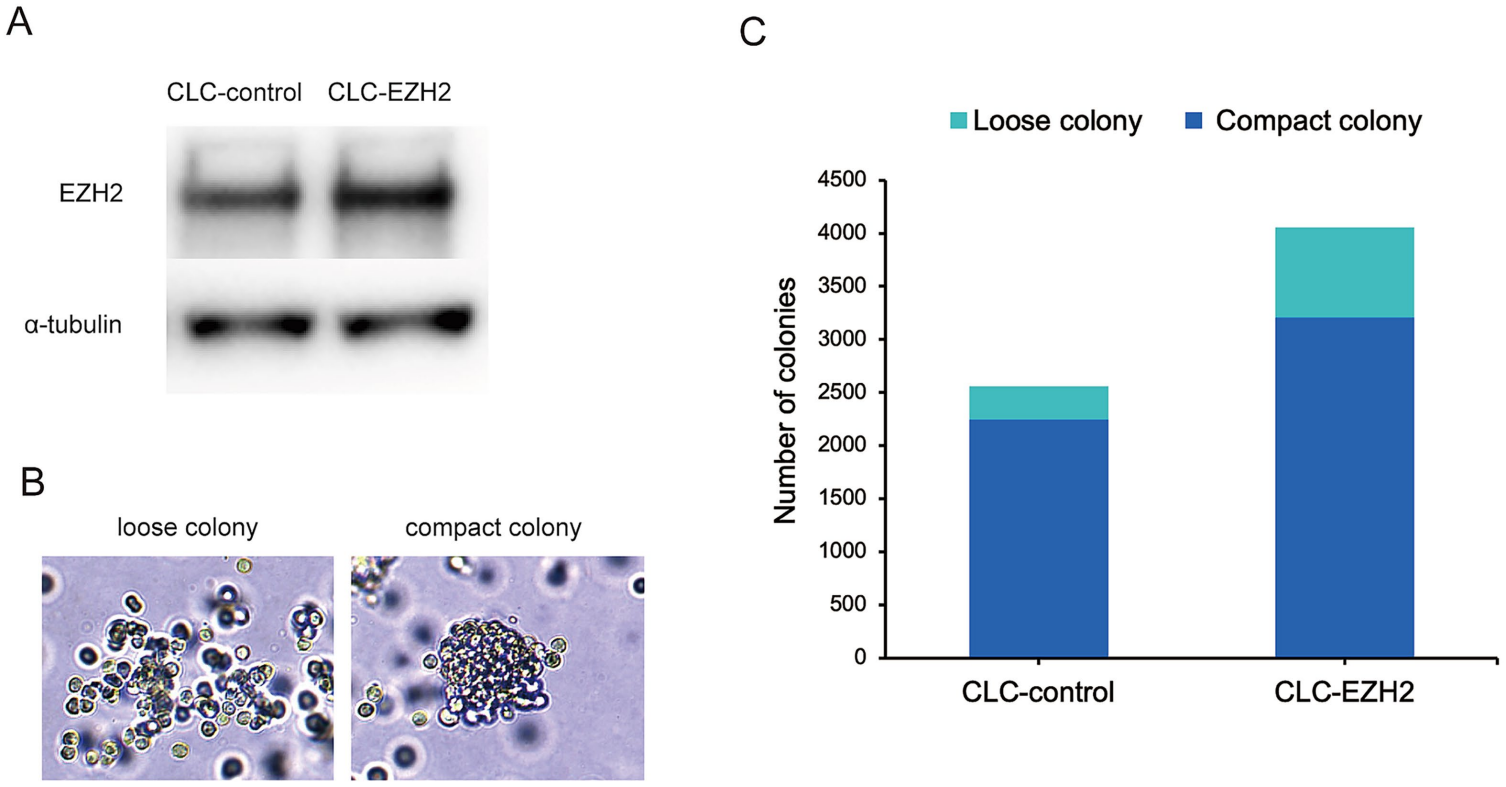
**(A)** Expression of EZH2 and *α*-tubulin in the canine T-cell lymphoma cell line CLC, its control cell line (CLC-control), and the EZH2 gene-transduced cell line (CLC-EZH2). **(B)** Morphology of loose and compact colonies formed by canine lymphoma cell lines on Methocult medium. **(C)** The figure shows the initial tally from the first of three independent experiments. Loose and compact colonies were counted separately. Compared with CLC-control cells, CLC-EZH2 cells exhibited significantly higher numbers of compact colonies (*p* = 0.03) and total colonies (*p* = 0.01), whereas no significant difference was observed in loose colonies.

A single band corresponding to EZH2 was detected in the cell line at a predicted molecular weight of 95 kDa (Figure 1A). A single band corresponding to *α*-tubulin was also detected in the cell line at a predicted molecular weight of 55 kDa as a control (Figure 1A). Western blot analysis showed enhanced expression following *EZH2* gene introduction.

### 3.2 EZH2 gene introduction increases colony formation ability in MethoCult medium

Figure 1B shows the morphology of loose and compact colonies formed in MethoCult medium, which assesses clonogenic growth under semi-solid conditions. Loose colonies may represent loosely aggregated cell clusters with a tendency toward cell death indicating poor viability, whereas compact colonies represent compact aggregates of viable, proliferative cells; however, it remains unclear whether the loose phenotype is intrinsic or secondary to culture conditions. Colony formation was evaluated by counting loose and compact colonies in methylcellulose cultures. The mean ± standard deviation of loose colony counts were 527.7 ± 136.5 for the CLC-control cell line and 787.0 ± 130.3 for the CLC-EZH2 cell line, with no statistically significant difference between groups (*p* = 0.24). In contrast, compact colony counts were significantly higher in CLC-EZH2 cells (3341.3 ± 146.5) compared with CLC-control cells (2511.0 ± 218.5; *p* = 0.03). Consistently, the total colony count was significantly increased in the CLC-EZH2 cell line (4128.3 ± 34.9) relative to the CLC-control cell line (3038.7 ± 252.2; *p* = 0.01). Figure 1C shows the initial colony counting results from the first experiment.

To further assess the effects of EZH2 overexpression while accounting for colony type and within-well dependency, colony counts were additionally analyzed using a mixed-effects two-way ANOVA with vector and colony type as fixed effects and well as a random effect. Analysis of raw colony counts demonstrated a significant main effect of EZH2 overexpression, with *post hoc* comparisons confirming a significant increase in compact colonies, whereas no significant difference was observed in loose colonies. Following log-transformation to stabilize variance, the effect of EZH2 overexpression did not reach statistical significance in either colony type, although a trend toward increased colony formation was observed. Together, these analyses indicate that EZH2 overexpression increases absolute colony numbers, particularly compact colonies, while the relative magnitude of this effect is modest when variance is taken into account.

### 3.3 EZH2 gene introduction decreased the proportion of apoptotic cells

**Figure 2:**
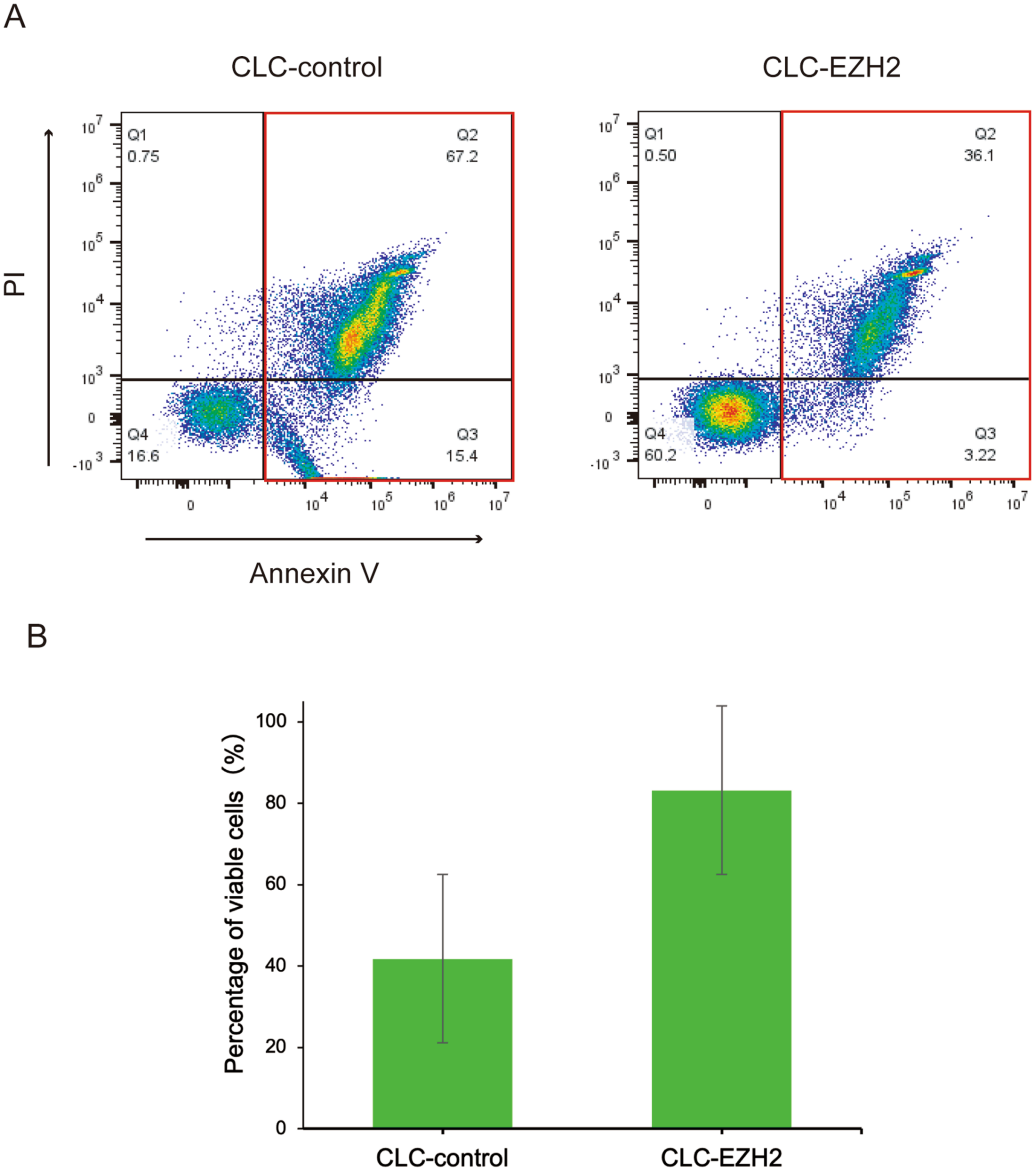
**(A)** Flow cytometry results for the Annexin V assay in CLC-control and CLC-EZH2. Annexin V-positive (apoptotic) cells are gated. The figure shows results representing four independent experiments. **(B)** Percentage of viable cells in CLC-control and CLC-EZH2. The figure shows the average of four independent experiments. The percentage of viable cells in CLC-EZH2 is significantly higher compared to CLC-control (*p* = 0.019).

Figure 2A shows the flow cytometry results for the Annexin V assay in CLC-control and CLC-EZH2. Viable cell numbers were significantly increased in CLC-EZH2 cells compared with control cells (41.8 ± 9.5 vs. 83.2 ± 8.9, *p* = 0.019; Figure 2B). The mean percentage of early apoptotic cells was 16.7% for the CLC-control cell line and 1.7% for the CLC-EZH2 cell line (*p* = 0.04). The mean percentage of late apoptotic cells was 46.6% for the CLC-control cell line and 25.7% for the CLC-EZH2 cell line (*p* = 0.63). Analysis of the four measurements revealed that the CLC-EZH2 cell line had a significantly higher proportion of viable cells and a lower proportion of early apoptotic cells compared to the CLC-control cell line. Furthermore, although not statistically significant, the CLC-EZH2 cell line tended to have a higher proportion of late apoptotic cells than the CLC-control cell line.

## 4 Discussion

Given the apparent importance of the function of the epigenetic regulator EZH2, we investigated the effects of further enhancing EZH2 activity. In canine T-cell lymphoma cell lines, EZH2 expression was detected, and enforced that the overexpression of EZH2 resulted in increased colony-forming ability and suppression of apoptosis. The expression of EZH2 and its association with tumorigenesis have been reported in numerous human tumors, including lymphoma; however, in dogs, although EZH2 expression has been described in several tumors, its direct association with tumorigenesis remains unclear (5).

In the present study, we were unable to elucidate the precise molecular mechanisms by which EZH2 suppresses apoptosis. While a number of studies have reported relevant findings, our understanding of the precise molecular mechanisms remains incomplete. Notably, chemical inhibition or depletion of EZH2 has been reported to induce apoptosis in both p53-proficient and p53-deficient cancer cells (10, 11). These findings suggest that EZH2 may regulate apoptosis through multiple pathways rather than through a single, linear mechanism. These pathways of apoptosis suppression are multi-faceted and include the downregulation of tumor suppressor genes such as *p53* and *GSK-3β* with the concurrent upregulation of oncogenes, such as *STAT3* and *NOTCH1* (12, 13). It is understood that this dual regulatory capacity contributes to increased cell survival and proliferation while also helping maintain the stem-like properties in tumor cells, which would in turn promote tumorigenesis and its progression (14). The contrasting fact that *EZH2* can also have a tumor suppressive role due to suffering loss-of-function mutations in certain hematological human tumors, illustrates a complex context-dependent function that requires further research to elucidate its role in CLC cell lines (13, 14).

Previous studies have reported that the *MYC* gene is highly expressed in the CLC cell line (8). Given that activation of the MYC-miRNA-EZH2 feed-forward loop as well as the MYC-EZH2-CDKN1C axis has been described in lymphomas, it is possible that CLC cells harbor such oncogenic regulatory networks (15, 16). In addition, the CLC cell line exhibits hyperphosphorylation of pRb (17). Since EZH2 has been reported to function downstream of the pRB-E2F pathway, these alterations may be mechanistically interconnected (18).

*EZH2* is known to harbor gain-of-function hotspot mutations, which are observed in a subset of human lymphomas (3, 7). These mutations can result in increased epigenetic heterogeneity, global reprogramming, and differentiation blockade, among other important oncogenic driving actions (19, 20). Although we were unable to assess the presence of such mutations in this study, it is plausible that similar *EZH2* mutations may also exist in canine lymphoma.

In the medical field, therapeutic agents targeting EZH2 exist, some currently undergoing different clinical trial phases to treat relapsing or refractory lymphomas (21). At the cellular level, studies with human-derived cells have reported that EZH2 confers resistance to apoptosis in cancer cells, one of the mechanisms being preventing specific gene expressions that would normally activate as lymphocytes complete maturation, thus driving lymphomagenesis (10, 21).

In Methocult medium, *EZH2* gene-transduced canine lymphoma cells exhibited significantly higher colony-forming capacity than control cells. This suggests that *EZH2* introduction may be associated with maintaining or enhancing the clonogenic capacity of cultured cells in canine lymphoma cell lines, as has already been demonstrated in mouse-derived hematopoietic cells (22) and human B-cell lymphoma cultured cells (23). However, whether this increase in colony formation directly reflects enhanced self-renewal capacity remains to be determined and warrants further investigation, including the analysis of stemness-associated markers.

We hypothesized that differences in apoptosis underlie the phenotypic changes observed in canine lymphoma cells cultured in MethoCult medium. To test this, we performed an Annexin V assay using flow cytometry. Compared to control cells, the *EZH2*-transduced canine lymphoma cell line exhibited significantly higher survival rates and a markedly lower proportion of apoptotic cells. Based on this result, it is considered that the introduction of the *EZH2* gene suppressed apoptosis in cultured canine lymphoma cells, thereby enhancing tumorigenesis by inducing changes in their proliferative capacity and survival.

In the present study, we attempted to introduce *EZH2* gene into three canine lymphoma cell lines; however, successful overexpression was achieved in only one cell line. Because the biological functions of EZH2 may differ depending on the cell line or individual case, further investigation will be required to clarify its context-dependent roles in canine lymphoma. In addition, the genetic profile of CLC cells has not yet been fully characterized, which should also be considered a limitation of the present study. Furthermore, because the analyses were performed using only a single cell line, the *in vivo* behavior and biological relevance of *EZH2* overexpression remain unclear. The present findings therefore represent only one aspect of the role of EZH2 in lymphoma, and further studies with additional experimental data will be required to clarify its broader biological significance.

To our knowledge, this is the first study evaluating phenotypic changes following *EZH2* gene introduction in canine lymphoma. Therefore, EZH2 may represent a potential novel molecular target for canine lymphoma treatment. As documented for human cell lines, we found that altered EZH2 expression in canine lymphoma cell lines also induces changes in the phenotype of cultured cells. The altered proliferation and survival capacity due to suppressed apoptosis is a critical factor related to tumor malignancy and prognosis. In conclusion, our findings then suggest that lymphoma cells may acquire resistance to cell death through the expression of EZH2. Although the precise mechanisms underlying EZH2-mediated suppression of apoptosis remain unclear, inhibition of EZH2 may represent a promising therapeutic strategy that targets epigenetic regulation to induce apoptosis in lymphoma cells.

## Data Availability

The raw data supporting the conclusions of this article will be made available by the authors, without undue reservation.
